# Initial sightings and derby data from the red lionfish invasion (*Pterois volitans*) (Scorpaeniformes: Scorpaenidae) in Barbados

**DOI:** 10.3897/BDJ.7.e38219

**Published:** 2019-08-29

**Authors:** Julian Walcott, Caroline Bissada, Hazel A Oxenford

**Affiliations:** 1 Centre for Resource Management and Environmental Studies, University of the West Indies, Cave Hill Campus, Cave Hill, Barbados Centre for Resource Management and Environmental Studies, University of the West Indies, Cave Hill Campus Cave Hill Barbados; 2 East Coast Conservation Organisation (ECCO) Inc., Bathsheba, Barbados East Coast Conservation Organisation (ECCO) Inc. Bathsheba Barbados

**Keywords:** lionfish, *Pterois volitans*, Barbados, occurrence, observation

## Abstract

**Background:**

Native to the Indo-Pacific region, the lionfish (*Pterois volitans* and *P. miles*) has been classified as an alien invasive species which has rapidly invaded the North-western Atlantic and the Caribbean. The primary concerns regarding lionfish pertain to their broad diet, general habitat use and their potential threat on fisheries resources, native fish communities and human health. Away from natural predators, lionfish populations can easily become established and pose a serious threat to local fish species and ecosystem functioning. The first confirmed sighting of the red lionfish (*Pterois volitans*) in Barbados was in November 2011. Throughout 2012, fishermen and recreational divers were encouraged to report sightings of lionfish via an established hotline. Where possible, sightings were confirmed by way of the fish being captured and handed over to the Barbados Fisheries Division or the East Coast Conservation Organisation (ECCO) Inc. (an environmental NGO) for confirmation. In addition to confirmation, biological data (such as length, weight, sex and maturity) were also collected. Genetic research conducted on confirmed specimens collected between 2011-2013 identified the presence of only one species, *P. volitans*. Since the first confirmed sighting of the red lionfish in Barbados, it is believed that population numbers have steadily increased. One of the methods utilised in Barbados to control this alien invasive lionfish species is that of an annual derby. On 5 and 6 December 2015 and 13 November 2016, teams of divers (both free divers and SCUBA divers) took to the local waters to hunt for and kill lionfish. Caught lionfish were landed at scoring stations to be counted and allowed for the collection of basic biological data (such as length, weight, sex and maturity). In addition to biological data, teams (observers) also provided information on dive sites (locations) and associated geographic information (i.e. GPS coordinates), where available.

**New information:**

These two datasets, initial sightings (2012) and derby data (2015 and 2016), present the first collected data for the red lionfish (P. volitans) in Barbados. The two datasets are occurrence datasets which document the identification of >1500 lionfish removed from the waters of Barbados between 2011 and 2016.

## Introduction

Native to the Indo-Pacific region, it is believed that the aquarium trade facilitated the introduction of lionfish (*Pterois volitans* [Linnaeus, 1758] and *Pterois miles* [Bennet, 1828]) to the United States of America (USA) (Morris et al. 2009, Albins 2013). Documentation of lionfish in the wild (i.e. via escape) dates back to 1985 (Morris 2012)off Florida and marks the beginning of one of the most rapid marine finfish invasions in history throughout the North-western Atlantic and the Caribbean (Morris et al. 2009, Albins 2013). By 2002, the invasion had progressed northwards along the east coast of the USA, reaching as far as New Jersey and eastwards to Bermuda (Morris et al. 2009). By 2012, the invasion had progressed southwards from its original location with documented cases of confirmed lionfish sightings throughout the Gulf of Mexico, Central America, South America and the Caribbean (Morris 2012). Barbados was one of the last Caribbean islands to be invaded, with the first confirmed sighting in November 2011 (Oxenford and Vallès 2014). Prior to this invasion, there have been no major incidences of marine invasive species affecting Caribbean coral reefs.

The primary concerns regarding lionfish pertained to their broad diet, general habitat use and their potential threat on fisheries resources, native fish communities and human health (Morris et al. 2009, Morris 2012). Lionfish may live for decades and reach sizes exceeding 47 cm (19 in), they become sexually mature in less than a year and reproduction can occur throughout the entire year (approx. every 4 days) (Morris 2012). Lionfish inhabit all marine habitats types (e.g. seagrass beds, coral and artificial reefs) and depths (from the shoreline to over 300 m or 100 ft) (Morris 2012). Away from their natural predators and in new environments where prey initially fail to recognise them as a threat, lionfish populations could easily become established and pose a serious threat to local fish species (including commercially important species) and ecosystem functioning (Albins 2013). In addition, the possession of 18 venomous spines (Morris et al. 2009) could lead to negative socioeconomic impacts by way of envenomations of fishermen, divers and recreational beach users (Morris 2012).

In recognition of the imminent arrival of lionfish, the Biodiversity Working Group of Barbados drafted a lionfish response plan (Brathwaite et al. 2011). For the first year of the Barbados invasion, all reported sightings were recorded (i.e. Barbados lionfish sightings database) to track the invasion (Oxenford and Vallès 2014). Genetic research conducted on collected samples (2011-2013) revealed a single species lionfish invasion (i.e. *P. volitans*) in Barbados (Sealy et al. 2014). By 2015, the lionfish had become established and numbers had increased to levels where mitigation measures, such as derbies, were deemed appropriate. Derbies, one of the conservation measures used to control populations of lionfish, represent organised events to remove as many lionfish as possible by teams or individuals, with the added benefit of their receiving prizes for performances. Derbies also act to engage stakeholders, provide education and awareness to the public and allow for the collection of important ecological data (Morris 2012). Two national lionfish derbies were held in Barbados in 2015 and 2016, providing the opportunity for the collection of basic biological data such as length, weight, sex and maturity and thus resulted in the creation of the Barbados lionfish derby database.

## Project description

### Title

Tracking the red lionfish invasion in Barbados

### Personnel

Julian Walcott, Caroline Bissada, Hazel Oxenford

### Study area description

**Figure 1.**
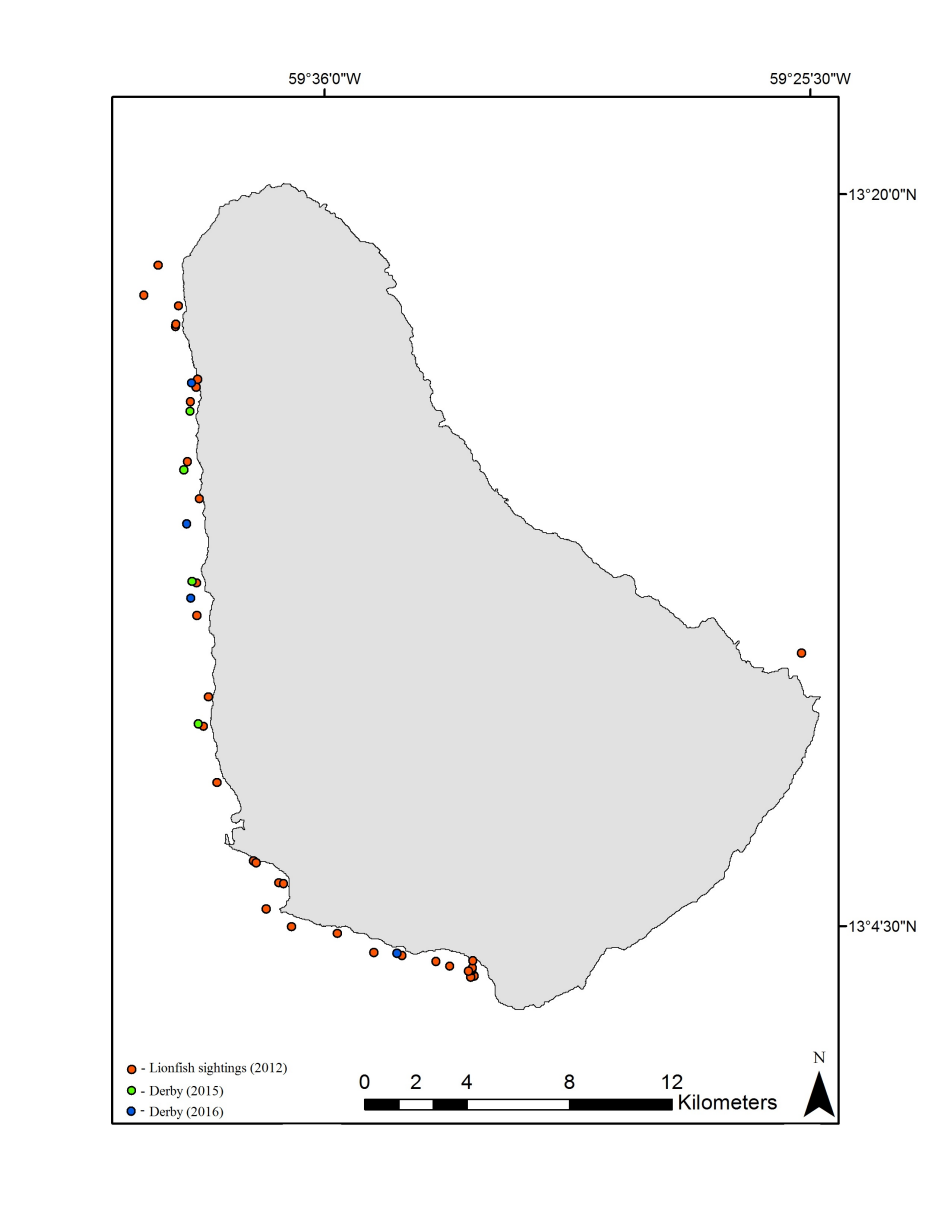
Confirmed lionfish (*Pterois volitans*) sightings and derby removals in Barbados.

**Figure 2.**
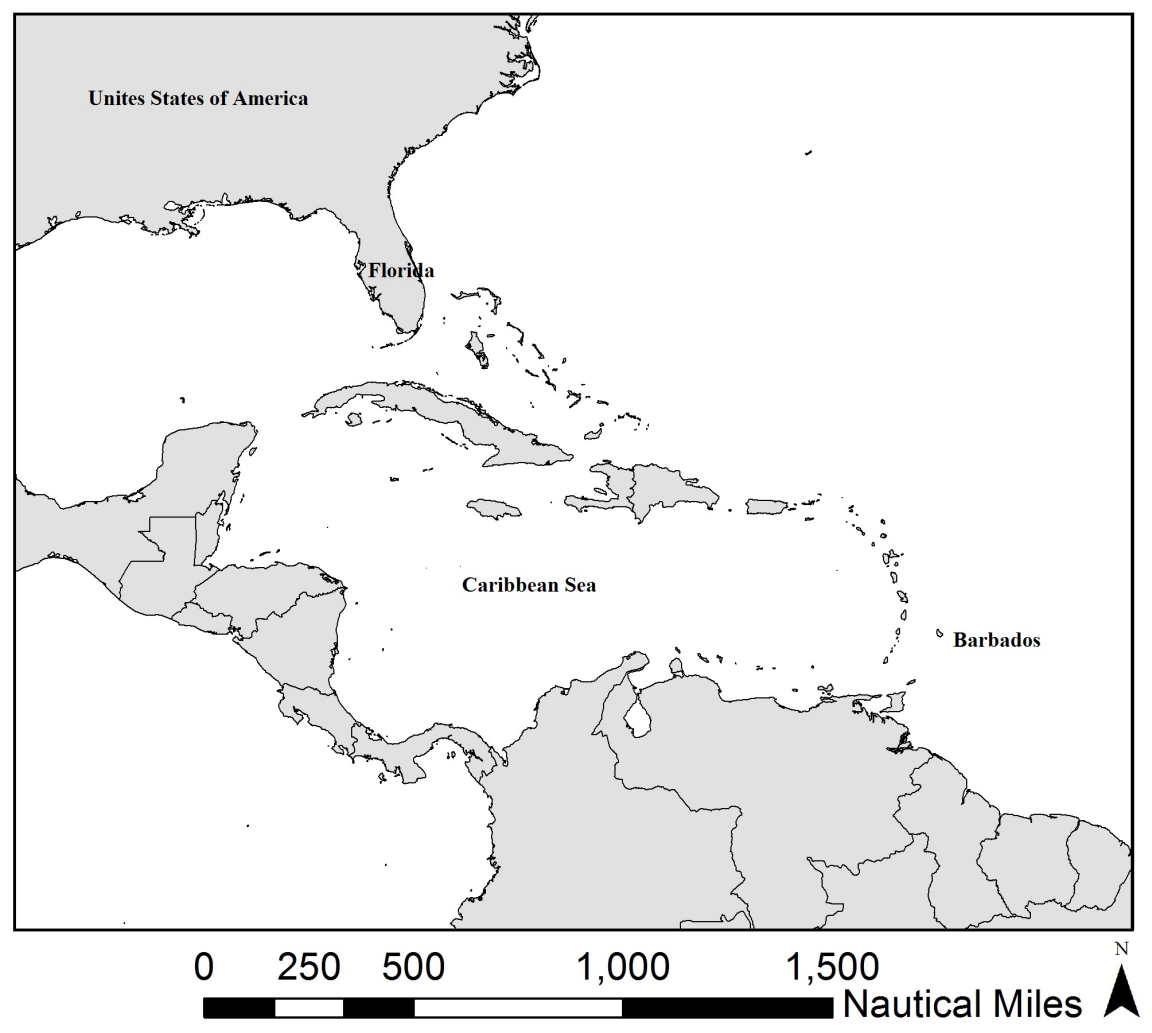
Caribbean Region

Barbados (Fig. 1) is a small Caribbean island (166 square miles), located to the east of the Caribbean island chain (Fig. 2), with a narrow insular shelf. Key marine habitats include fringing reefs (primarily along the west coast), patch reefs and a bank reef which runs parallel to shore.

### Funding

GBIF: BID-CA2016-0013-SMA

## Sampling methods

### Sampling description

The first confirmed sighting of a lionfish in Barbados was in November 2011. Throughout 2012, persons (i.e. fishermen and recreational divers) were encouraged to report sightings of lionfish. Where possible, sightings were confirmed by way of the fish being captured and handed over to the relevant persons from the Barbados Fisheries Division (Government agency) or the East Coast Conservation Organisation (ECCO) Inc. (an environmental NGO) for confirmation. In addition to confirmation, biological data (such as length, weight, sex and maturity) were also collected. Collected data were subsequently shared with the University of the West Indies for data analysis and management.

On 5 and 6 December 2015 and 13 November 2016, teams of divers (both free divers and SCUBA divers) took to the local waters to hunt for and kill lionfish, during organised derby events. Caught lionfish were landed at a scoring station to be counted and to allow for the collection of basic biological data (such as length, weight, sex and maturity). In addition to biological data, teams (observers) also provided information on dive sites (locations) and associated geographic information (i.e. GPS coordinates), where available (unfortunately, fish collected at individual dive sites were not separated on the boat, thus making it difficult to assign locations to many of the landed fish). Collected data were subsequently shared with the University of the West Indies for data analysis and management.

## Geographic coverage

### Description

Barbados

### Coordinates

12.989 and 13.384 Latitude; -59.705 and -59.365 Longitude.

## Taxonomic coverage

### Taxa included

**Table:** 

Rank	Scientific Name	
species	*Pterois volitans* (Linnaeus, 1758)	

## Traits coverage

### Data coverage of traits

PLEASE FILL IN TRAIT INFORMATION HERE

## Temporal coverage

### Notes

2012-01-01 through 2012-12-31, 2015-12-05 through 2015-12-06, 2016-11-13 through 2016-11-13.

## Usage rights

### Use license

Creative Commons Public Domain Waiver (CC-Zero)

### IP rights notes

This work is licensed under a Creative Commons Attribution (CC-BY) 4.0 License .

## Data resources

### Data package title

CERMES Barbados Lionfish Sightings 2012, CERMES Barbados Lionfish Derby Data

### Alternative identifiers

8f6d3a0d-10d2-44e3-8612-be219376c860; http://ipt.vertnet.org:8080/ipt/resource?r=cermes_sightings2012 7904180c-36c9-40ca-aed2-463e36a04df7; http://ipt.vertnet.org:8080/ipt/resource?r=cermes_lionfishderby

### Number of data sets

2

### Data set 1.

#### Data set name

CERMES Barbados Lionfish Sightings 2012

#### Data format

Darwin Core

#### Number of columns

56

#### Download URL

https://www.gbif.org/dataset/8f 6d3a0d-10d2-44e3-8612-be219376c860

#### Description

This dataset provides information on the first year of the lionfish invasion in Barbados. The first confirmed sighting of a lionfish in Barbados was in November 2011. Throughout 2012, persons were encouraged to report sightings of lionfish. Where possible, sightings were confirmed by way of the fish being captured and handed over to the relevant persons for confirmation. In addition to confirmation, biological data (such as length, weight, sex and maturity) were also collected. This dataset provides basic information for the confirmed sightings, such as date, time, habitat and activity where possible.

**Data set 1.** 

Column label	Column description
type	The nature or genre of the resource
modified	The most recent date-time on which the resource was changed
language	The language of the resource
licence	A legal document giving official permission to do something with the resource
rightsHolder	A person or organisation owning or managing rights over the resource
accessRights	Information about who can access the resource or an indication of its security status
bibliographicCitation	A bibliographic reference for the resource as a statement indicating how this record should be cited (attributed) when used
references	A related resource that is referenced, cited or otherwise pointed to by the described resource
institutionCode	The name (or acronym) in use by the institution having custody of the object(s) or information referred to in the record
collectionCode	The name, acronym, coden or initialism identifying the collection or dataset from which the record was derived
datasetName	The name identifying the dataset from which the record was derived
ownerInstitutionCode	The name (or acronym) in use by the institution having ownership of the object(s) or information referred to in the record
basisOfRecord	The specific nature of the data record
informationWithheld	Additional information that exists, but that has not been shared in the given record
occurrenceID	An identifier for the Occurrence (as opposed to a particular digital record of the occurrence). In the absence of a persistent global unique identifier, construct one from a combination of identifiers in the record that will most closely make the occurrenceID globally unique
catalogNumber	An identifier (preferably unique) for the record within the dataset or collection
recordedBy	A list (concatenated and separated) of names of people, groups or organisations responsible for recording the original Occurrence
individualCount	The number of individuals represented present at the time of the Occurrence
behaviour	The behaviour shown by the subject at the time the Occurrence was recorded
establishmentMeans	The process by which the biological individual(s) represented in the Occurrence became established at the location
occurrenceStatus	A statement about the presence or absence of a Taxon at a Location
organismID	An identifier for the Organism instance (as opposed to a particular digital record of the Organism)
eventDate	The date-time or interval during which an Event occurred. For occurrences, this is the date-time when the event was recorded
eventTime	The time or interval during which an Event occurred
startDayOfYear	The earliest ordinal day of the year on which the Event occurred (1 for 1 January, 365 for 31 December, except in a leap year, in which case it is 366)
endDayOfYear	The latest ordinal day of the year on which the Event occurred (1 for 1 January, 365 for 31 December, except in a leap year, in which case it is 366)
year	The four-digit year in which the Event occurred, according to the Common Era Calendar
month	The ordinal month in which the Event occurred
day	The integer day of the month on which the Event occurred
verbatimEventDate	The verbatim original representation of the date and time information for an Event
habitat	A category or description of the habitat in which the Event occurred
higherGeography	A list (concatenated and separated) of geographic names less specific than the information captured in the locality term
continent	The name of the continent in which the Location occurs
islandGroup	The name of the island group in which the Location occurs
island	The name of the island on or near which the Location occurs
country	The name of the country or major administrative unit in which the Location occurs
countryCode	The standard code for the country in which the Location occurs
locality	The specific description of the place
verbatimLocality	The original textual description of the place
minimumDepthInMetres	The lesser depth of a range of depth below the local surface, in metres
maximumDepthInMetres	The greater depth of a range of depth below the local surface, in metres
decimalLatitude	The geographic latitude (in decimal degrees, using the spatial reference system given in geodeticDatum) of the geographic centre of a Location
decimalLongitude	The geographic longitude (in decimal degrees, using the spatial reference system given in geodeticDatum) of the geographic centre of a Location
geodeticDatum	The ellipsoid, geodetic datum or spatial reference system (SRS) upon which the geographic coordinates given in decimalLatitude and decimalLongitude are based
georeferenceVerificationStatus	A categorical description of the extent to which the georeference has been verified to represent the best possible spatial description
scientificName	The full scientific name, with authorship and date information, if known
higherClassification	A list (concatenated and separated) of taxa names terminating at the rank immediately superior to the taxon referenced in the taxon record
kingdom	The full scientific name of the kingdom in which the taxon is classified
phylum	The full scientific name of the phylum or division in which the taxon is classified
class	The full scientific name of the class in which the taxon is classified
order	The full scientific name of the order in which the taxon is classified
family	The full scientific name of the family in which the taxon is classified
genus	The full scientific name of the genus in which the taxon is classified
specificEpithet	The name of the first or species epithet of the scientificName
taxonRank	The taxonomic rank of the most specific name in the scientificName
nomenclaturalCode	The nomenclatural code (or codes in the case of an ambiregnal name) under which the scientificName is constructed

### Data set 2.

#### Data set name

CERMES Barbados Lionfish Derby Data

#### Data format

Darwin Core

#### Number of columns

43

#### Download URL

https://www.gbif.org/dataset/7904180c-36c9-40ca-aed2-463e36a04df7

#### Description

Since its first confirmed sighting in November 2011, it is believed that the lionfish population has steadily increased. One of the methods utilised in Barbados to control the alien invasive lionfish is that of an annual derby. On 5 and 6 December 2015 and 13 November 2016, teams of divers (both free divers and SCUBA divers) took to the local waters to hunt for and kill lionfish. Caught lionfish were landed at a scoring station to be counted and to allow for the collection of basic biological data (such as length, weight, sex and maturity). In addition to biological data, teams (observers) also provided information on dive sites (locations) and associated geographic information (i.e. GPS coordinates) where available. This event was a Reef Environmental Education Foundation (REEF) sanctioned derby event (see http://www.reef.org/lionfish/events)

**Data set 2.** 

Column label	Column description
type	Identifies the species in the dataset
modified	The most recent date-time on which the resource was changed
language	A language of the resource
licence	A legal document giving official permission to do something with the resource
accessRights	Information about who can access the resource or an indication of its security status
references	A related resource that is referenced, cited or otherwise pointed to by the described resource
institutionCode	The name (or acronym) in use by the institution having custody of the object(s) or information referred to in the record
collectionCode	The name, acronym, coden or initialism identifying the collection or dataset from which the record was derived
datasetName	The name identifying the dataset from which the record was derived
basisOfRecord	The specific nature of the data record
informationWithheld	Additional information that exists, but that has not been shared in the given record
occurrenceID	An identifier for the Occurrence (as opposed to a particular digital record of the occurrence)
catalogNumber	An identifier (preferably unique) for the record within the dataset or collection
recordedBy	recordedBy
individualCount	The number of individuals represented present at the time of the Occurrence
establishmentMeans	The process by which the biological individual(s) represented in the Occurrence became established at the location
occurrenceStatus	A statement about the presence or absence of a Taxon at a Location
organismID	An identifier for the Organism instance (as opposed to a particular digital record of the Organism)
eventDate	The date-time or interval during which an Event occurred
startDayOfYear	The earliest ordinal day of the year on which the Event occurred (1 for 1 January, 365 for 31 December, except in a leap year, in which case it is 366)
endDayOfYear	The latest ordinal day of the year on which the Event occurred (1 for 1 January, 365 for 31 December, except in a leap year, in which case it is 366)
year	The four-digit year in which the Event occurred, according to the Common Era Calendar
month	The ordinal month in which the Event occurred
day	The integer day of the month on which the Event occurred
verbatimEventDate	The verbatim original representation of the date and time information for an Event
higherGeography	A list (concatenated and separated) of geographic names less specific than the information captured in the locality term
continent	The name of the continent in which the Location occurs
islandGroup	The name of the island group in which the Location occurs
island	The name of the island on or near which the Location occurs
country	The name of the country or major administrative unit in which the Location occurs
countryCode	The standard code for the country in which the Location occurs
verbatimLocality	The original textual description of the place
scientificName	The full scientific name, with authorship and date information, if known
higherClassification	A list (concatenated and separated) of taxa names terminating at the rank immediately superior to the taxon referenced in the taxon record
kingdom	The full scientific name of the kingdom in which the taxon is classified
phylum	The full scientific name of the phylum or division in which the taxon is classified
class	The full scientific name of the class in which the taxon is classified
order	The full scientific name of the order in which the taxon is classified
family	The full scientific name of the family in which the taxon is classified
genus	The full scientific name of the genus in which the taxon is classified
specificEpithet	The name of the first or species epithet of the scientificName
taxonRank	The taxonomic rank of the most specific name in the scientificName
nomenclaturalCode	The nomenclatural code (or codes in the case of an ambiregnal name) under which the scientificName is constructed

## Additional information

CERMES. 2012. 2012 Barbados Lionfish Sightings. Centre for Resource Management and Environmental Studies, University of the West Indies, Cave Hill Campus, Cave Hill, Barbados.

ECCO Inc. 2016. 2015-16 Barbados Lionfish Derby Data. East Coast Conservation Organisation Incorporated, St. Joseph, Barbados.
